# Association between Neonatal Whole Blood Iron Content and Cytokines, Adipokines, and Other Immune Response Proteins

**DOI:** 10.3390/nu11030543

**Published:** 2019-03-04

**Authors:** Steffen U. Thorsen, Christian B. Pipper, Christina Ellervik, Flemming Pociot, Julie N. Kyvsgaard, Jannet Svensson

**Affiliations:** 1 Copenhagen Diabetes Research Center (CPH-DIRECT), Department of Paediatrics, Herlev Hospital, University of Copenhagen, Herlev Ringvej 75, 2730 Herlev, Denmark; flemming.pociot.01@regionh.dk (F.P.); juliekyvs@hotmail.com (J.N.K.); Jannet.Svensson@regionh.dk (J.S.); 2 Department of Public Health, Section of Biostatistics, University of Copenhagen, Copenhagen, Oester Farimagsgade 5, 1710 Copenhagen K, Denmark; pipper@sund.ku.dk; 3 Department of Production, Research, and Innovation, Region Zealand, Alleen 15, 4180 Sorø, Denmark; christina@ellervik.dk; 4 Department of Clinical Medicine, Faculty of Health and Medical Sciences, University of Copenhagen, Blegdamsvej 3B, 2200 Copenhagen N, Denmark; 5 Department of Laboratory Medicine, Boston Children’s Hospital, Harvard Medical School, 300 Longwood Avenue, Boston, MA 02115, USA; 6 Steno Diabetes Center Copenhagen, Niels Steensensvej, 2820 Gentofte, Denmark

**Keywords:** cytokines, adipokines, diabetes mellitus, type 1, iron, TREM1, human, C-reactive protein, mannose-binding lectin, infant, newborn

## Abstract

(1) Background: High iron associates with inflammation and type 1 diabetes (T1D). Iron is essential not only for neonatal development but also for infectious microorganisms. The neonatal immune system is immature, and innate immunity prevails before immunocompetence develops. (2) Methods: In 398 newborns from the Danish Newborn Screening Biobank, we examined if whole blood iron (WB-Iron) content were associated with cytokines, adipokines, C-reactive protein (CRP), and mannose-binding lectin (MBL) in non-infected healthy neonates, and if these associations differed in newborns who later developed T1D (cases) (*n* = 199). WB-Iron was quantified using laser ablation inductively coupled plasma mass spectrometry on the neonatal dried blood spots. For each analyte, the relative change (RC) in the mean level was modeled by robust log-normal regression. (3) Results: A one unit increase in neonatal WB-Iron was associated with a 38% decrease in mean interleukin (IL)-6 levels (0.62; 95% CI: 0.40–0.95, *p* = 0.03), and a 37% decrease in mean MBL levels (0.63; 95% CI: 0.41–0.95, *p* = 0.03), but was not statistically significant after correction for multiple testing. (4) Conclusions: In summary, we found that higher neonatal WB-iron content was inversely associated with IL-6 and MBL, which may increase susceptibility to infections.

## 1. Introduction

The neonatal immune system is immature, and innate immunity prevails before immunocompetence develops. Following birth and during early childhood, the immune system must rapidly develop to adapt to the infectious microorganisms in the extrauterine environment as well as to establish tolerance to abundant non-pathogenic antigens [1]. The innate immune system is important, especially during early life, and represents the first line of defense against pathogenic microbes [2]. As part of the innate immune system, mannose-binding lectin (MBL) binds to pathogens and activates the complement pathway [3]. Cytokines, which act as intercellular signaling molecules in the immune system, regulate not only the innate immune system (e.g., tumor necrosis factor alpha (TNFα), interleukin (IL)-1β, IL-6, IL-8, IL-10, IL-12) but also the adaptive system (IL-4, interferon gamma (IFNγ), and transforming growth factor beta 1 (active form) (TGFβ)).

Iron (Fe) is an essential trace element not only for neonatal development but also for infectious microorganisms. Sequestration of circulating iron prevents bacterial iron utilization [4]. This mechanism is regulated by hepcidin, which is increased in response to infection and inflammation and induced by IL-6, a pattern belonging to the innate immune system. Defects in hepcidin production are associated with iron overload disorder hemochromatosis (hepcidin deficiency), anemia of chronic inflammation or anemia of iron deficiency (hepcidin excess) [5,6]. Sequestration of iron also occurs independently of hepcidin, in response to TNFα, IL-1β, and IFNγ [7]. In adults, iron overload is associated with increases in complement C3 and C-reactive protein (CRP), an acute phase reactant in inflammation and infection and regulated by IL-6 [8]. The hypothesis of a bidirectional cross-talk between iron and the immune system has emerged recently, but this interaction is still poorly understood [9,10]. Results from each end of the iron status spectrum, for example, severe deficiency (iron-deficiency anemia) and extreme overload (hereditary hemochromatosis (HH) and beta-thalassemia major), have indicated changes in lymphocyte proliferation and cytokine profiles [9,10].

Iron overload is associated with diabetes in adults and children [11,12,13]. The mechanism involved is most likely due to intracellular iron accumulation in beta cells, generation of oxidative stress, and apoptosis [14,15]. However, increased iron is also associated with type 2 diabetes [12], which is characterized by insulin resistance combined with beta-cell failure, and adipokines (e.g., adiponectin, leptin) function as intercellular molecules between adipose tissue and pancreas [16].

Till date, no studies have examined if a link between physiological levels of circulating iron measures and a variety of cytokines, adipokines or immune response proteins exist in a non-infected healthy neonatal population, which could provide new insights regarding the role of iron in immunological health and disease. Manipulation of iron status may potentially represent a simple way of (re)-gaining immunological homeostasis [17].

Utilizing neonatal dried blood spots (NDBS) from the unique Danish Newborn Screening Biobank (DNSB) [18], we aimed to elucidate if: i. neonatal whole blood iron (WB-Iron) content was associated with cytokines, adipokines, and other proteins involved in the immune response (CRP, MBL, and soluble triggering receptor expressed on myeloid cells-1 (sTREM-1)) and ii. case status (neonates that later develops childhood type 1 diabetes (T1D) versus neonates that do not develop childhood T1D) was an effect modifier of the latter associations.

## 2. Materials and Methods 

### 2.1. Overview of Study Design and Sampling

This population-based study consists of a case-cohort sample from the DNSB [18], which is located at Statens Serum Institute, Copenhagen, Denmark. Since 1981, the dried blood spots (DBS) have been stored at −20 °C/−4 °F in the DNSB, and this biobank covers close to 100% of the Danish population born since 1982 [19].

Originally, the sample consisted of triplets (1:2) [20], but only duplets (1:1) had whole blood iron content measured [11], which restricts this study to a 1:1 sampling. Cases and controls were matched on date-of-birth. Participants were born between January 1991 and November 1998 and cases developed T1D before the age of 16 years (detailed information about the study sample is given elsewhere [11,20]). Briefly, T1D diagnosis was classified according to World Health Organization International Classification of Diseases (ICD) criteria using ICD-8 (codes: 249 and 250) and ICD-10 (codes: DE10.x–14.x) (ICD-9 has not been used in Denmark). Cases have been thoroughly validated by the Danish Registry of Childhood and Adolescent Diabetes (DanDiabKids) [21]. 

**Figure 1:**
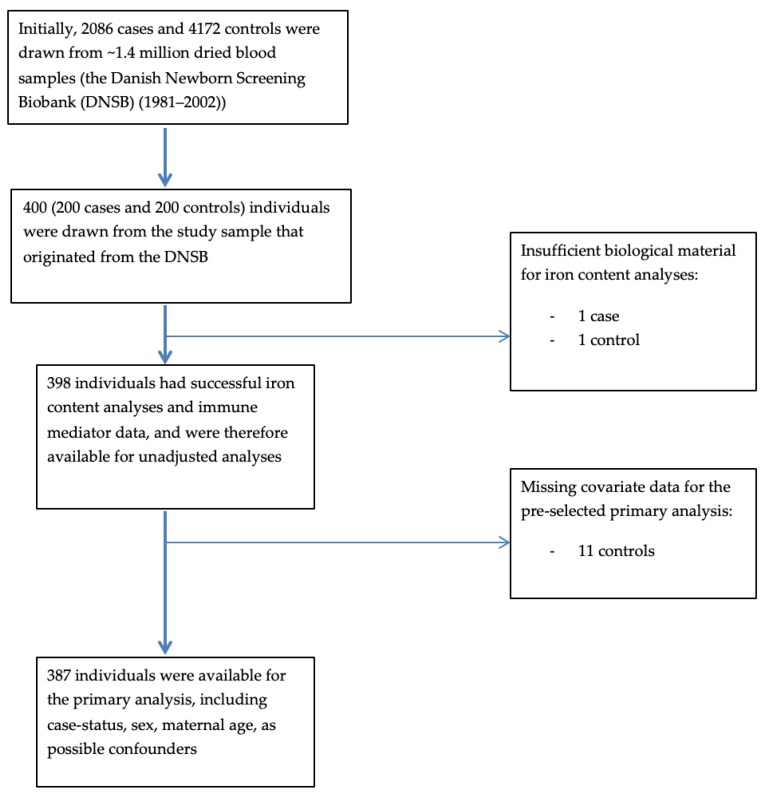
Flow chart of participants included in the study and different models.

We had complete exposure and outcome data on 398 participants (199 cases and 199 controls) (see a flow chart of participants in Figure 1).

### 2.2. Exposure Assessment 

#### 2.2.1. Assessment of Whole Blood Iron Content

The samples were analyzed for iron (^56^Fe) using laser ablation inductively coupled plasma mass spectrometry (LA-ICP-MS). The samples were also analyzed for potassium (^39^K) in the same run to control for blood volume on the NDBS and the hematocrit value in the newborn [22]. All of the samples were analyzed together to minimize operational variation [11,23]. 

The LA-ICP-MS measures 12 consecutive points on each NDBS, which increases the accuracy of the neonatal WB-Iron content (total sum/12) [24,25]. Further, LA-ICP-MS measures the ion intensity in counts per second, which is proportional to the iron concentration. The unit of measurement is total iron ion content in capillary whole blood relative to the total potassium ion content in capillary whole blood [11].

During the study period (1991–1998), the same filter paper had been used, thereby eliminating the risk of including filter paper with different iron-binding capacity.

#### 2.2.2. Other Variables 

A number of basic variables and possible confounders were available. These variables and their coding are presented in Table 1.

### 2.3. Outcome Assessment

Fourteen analytes were quantified on the 3.2 mm diameter NDBS using a multiplexed sandwich immunoassay, based on flowmetric Luminex xMAP^®^ technology. These were: i. cytokines: IL-1β, IL-4, IL-6, IL-8, IL-10, IL-12(p70), IFNγ, TNFα, TGFβ; ii. energy hormones/adipokines: leptin and adiponectin; and iii. CRP and MBL involved in innate immunity, and sTREM-1 belonging to the immunoglobin superfamily. Biomarker analyses are described in depth elsewhere [26]. In all assays, matched pairs were analyzed together to avoid operational variation [23]. 

Quality control of the analysis was made using mouse IL-6 as an internal analyte added to the extraction buffer to detect pipetting errors, and biotinylated beads to detect signal errors [27]. Calibration curves were used on each plate together with one high and two low controls. Samples, calibrators, and controls were analyzed in duplicates.

### 2.4. Statistical Analysis

For each analyte, the relative change (RC) in the mean level by one unit increase in neonatal WB-Iron content was modeled by a robust log-normal model regression, which takes into account: i. that measurements are potentially both left and right censored; and ii. correlation within immunoassay. To account for correlation within immunoassay, an inference was based on a working independence generalized estimation equation (GEE) approach. 

The simultaneous evaluation of neonatal WB-Iron content on all analytes was done using the model stacking approach detailed in Pipper et al. [28]. Subsequent adjustment for multiple testing and familywise 95% confidence bands (95% CI) were calculated using the single step procedure by Hothorn et al. [29]. GEE estimates of mean ratios and accompanying confidence limits were calculated on a log-scale and transformed back to the original scale.

Model selection was done prior to statistical analyses based on our previous work using these data: i. univariate models; and ii. primary adjusted models using possible confounders (covariates associated with neonatal WB-Iron content (sex, case status, and maternal age) (see Kyvsgaard et al. [11]).

Overall functional misspecification by including neonatal WB-Iron content as a trend (linear variable) was assessed by a lack-of-fit test. Specifically, we included a quadratic term of neonatal WB-Iron content and tested its significance by a robust Wald test.

Due to the fact that our study population consisted of neonates who later developed childhood T1D (*n* = 199) and those who did not develop T1D (*n* = 199), we also performed adjusted stratified analyses to examine if the relationship between analytes and WB-Iron content may be different between cases and controls. Further, if estimates differed between cases and controls, we examined this difference using the test of interaction (*p*-difference) [30].

We also performed post-hoc analyses to secure that the associations between neonatal WB-Iron content and IL-6 and leptin were independent of leptin and IL-6 levels, respectively. This was done due to previously described positive associations [31,32]. Due to the observed positive and u-shaped associations between these two analytes, in the present study, the adjustments were done using proper linear and non-linear (quadratic term) modeling (Figure S1). 

*P*-values were evaluated at a two-sided 5% significance level, and prior to our statistical analyses, we decided to include both the results with and without adjustment for multiple testing. 

All analyses were made using the statistical software package R version 3.5.1 (the R foundation for statistical programming, Vienna, Austria), the add-on packages survival, and multcomp.

### 2.5. Ethics

The study was performed in accordance with the Helsinki II Declaration. Furthermore, the study was approved by the Danish Ethical Committee (H-2-2014-007) and by the DNSB Steering Committee. According to Danish law, anonymous studies do not require further informed consent. 

## 3. Results

### 3.1. Basic Characteristics

Characteristics of the study population are presented in Table 1. 

Overall median (Q1, Q3) WB-Iron content was 1.75 (1.56, 1.95) units. When stratifying by case status, the median WB-Iron contents were 1.76 (1.59, 1.98) and 1.74 (1.54, 1.94) units for cases and controls, respectively. Median (Q1, Q3) concentrations, by case status, for the 14 analytes are presented in Table S1. Spearman’s correlation between the cytokines, adipokines, and other proteins involved in the immune response are depicted in Figure S2. 

### 3.2. Unadjusted Models

For one unit increase in neonatal WB-Iron content, there was a 38% decrease in mean IL-6 levels (0.62; 95% CI: 0.40–0.95, *p* = 0.03) and a 38% decrease in mean MBL levels (0.62; 95% CI: 0.41–0.95, *p* = 0.03) (Table 2). These associations did not remain statistically significant after correction for multiple testing (Table 3).

### 3.3. Adjusted Models

For one unit increase in neonatal WB-Iron content, a 38% decrease in mean IL-6 levels (0.62; 95% CI: 0.40–0.95, *p* = 0.03) and a 37% decrease in mean MBL levels (0.63; 95% CI: 0.41–0.95, *p* = 0.03) were found (Table 2). However, these associations were not statistically significant after correction for multiple testing (Table 3). In addition, we found no sign of non-linear associations. 

### 3.4. Sensitivity Analyses

When stratifying on case status and performing the adjusted models, we found the same directions of effect regarding MBL and IL-6, but naturally with a reduction in power. In cases, leptin was inversely associated with neonatal WB-Iron content (0.58; 95% CI: 0.36–0.92, *p* = 0.02) compared to (1.09; 95% CI: 0.78–1.51, *p* = 0.63) in controls (*p*-difference = 0.03). Furthermore, IL-1β was positively associated with neonatal WB-Iron content (2.01; 95% CI: 1.10–3.67, *p* = 0.02) but not in controls (1.14; 95% CI: 0.64–2.03, *p* = 0.67) (*p*-difference = 0.24). When corrected for multiple testing, the results were not significant (Table S2).

Our post-hoc analyses showed that the association between WB-iron and IL-6 was unaltered when leptin was included in the unadjusted model (independent effect) and vice versa. 

## 4. Discussion

In this study, we investigated the association between neonatal WB-Iron content and cytokines, adipokines, and other proteins involved in the immune response, and if this was different in neonates who later developed childhood T1D vs. those who did not. No previous study has investigated this association in neonates. Overall in adjusted models, we found inverse associations between WB-Iron content and circulating neonatal IL-6 and MBL. In newborns, who later developed T1D, we found an inverse association between WB-Iron levels and leptin, which was different from that in controls, and a positive association with IL-1β, which was not different from that in controls. Adjustment for multiple testing removed these associations. 

### 4.1. Comparison with Other Studies

The finding of an inverse association between neonatal WB-Iron content and circulating neonatal IL-6 levels is in line with murine studies that the peptide hormone hepcidin, which is up-regulated in response to higher iron levels, suppresses IL-6 gene expression upon inflammatory stimuli [33]. Newborns are at risk of severe infections, and this is due in large part to a naïve adaptive immune response [34]. Iron is needed for bacterial growth, and too much circulating neonatal iron may pose a risk in regards to providing a favorable milieu for bacterial growth [35]. Thus, these results may point to a modulatory effect of physiological high iron levels on IL-6 production possibly mediated through hepcidin. Though other murine studies have found that in vitro macrophage cytokine production, for example, IL-6 and TNFα, is caused by low intracellular iron levels [36].

Iron deficiency in acutely ill hospitalized children (*n* = 142, mean age = 3 years), in Malawi, has been linked with increased IL-6 production from lymphocytes examined ex vivo [37]. In contrast, a study in healthy Indonesian infants (*n* = 52, age range = 3–9 months) found no association between iron deficiency and ex vivo whole blood cytokine production, including IL-6 production [38], though this study was underpowered. 

IL-6 has been positively associated with an increased transferrin receptor density on hepatocytes favoring iron storage in the liver, an increase in ferritin synthesis, and a decrease in transferrin receptor synthesis [37]. In our study, higher neonatal WB-Iron content seems to decrease circulating IL-6, which in the light of the formerly mentioned effects of IL-6 on iron homeostasis makes sense regarding no need for further intracellular iron storage/accumulation, but at the expense of providing a substrate for microbial agent growth.

We also found an inverse association between neonatal WB-Iron content and circulating neonatal MBL levels. MBL is a protein of innate immunity, an acute phase protein, that activates the complement system and promotes phagocytosis [3]. Lower MBL levels in the neonatal period and up through childhood have been associated with higher risk and progression of severe infections [3], and higher MBL levels are found in children with newly diagnosed childhood T1D [39]. To our knowledge, no studies have examined the association between iron and MBL levels in a pediatric population, but a small study designed to examine the safety of five different intravenous iron preparations found no association between supra-physiological iron loading and MBL concentrations in adults [40]. In addition, it is important to mention that serum polymerization is critical for the biological activity of MBL, which we, unfortunately, have not been able to account for [3]. 

We also found an inverse relationship between neonatal WB-Iron content and circulating neonatal leptin levels, which seems only to be present in the case group. Leptin is a major regulator of appetite, energy homeostasis, metabolism, and may also influence the immune response, for example, a decrease in leptin levels is associated with an increase in infection susceptibility [31,41]. Our finding has also been demonstrated among in vitro, murine, and human studies [42]. The molecular mechanism behind this inverse association seems to be through regulation of leptin transcription by iron in the adipocytes [42]. Our research group has previously found a 26% reduction in leptin levels in newly diagnosed patients with childhood T1D compared to their siblings without childhood T1D [41] but have not been able to find differences in leptin levels between neonates that later develops childhood T1D and their controls [20]. Whether the inconsistency in the two latter study results is due to different/same iron distributions between groups and/or time-dependent effects remains unexamined. 

Noteworthy, leptin may also mediate the production of other cytokines, for example, IL-6, which adds an extra layer of complexity in deciphering which cytokine-pathways are iron-sensitive and which are influenced by other cytokines/immune mediators [31], but we found that the associations between neonatal WB-Iron content and IL-6 and leptin were unaltered after adjusting for leptin and IL-6 levels, respectively. 

Despite that iron overload is associated with increases in complement C3 and CRP in adults [8], we did not find associations between WB-Iron and CRP in neonates. This is most likely explained by either: i. WB-Iron content was not being sufficiently high, or ii. the duration of exposure had not been long enough to induce chronic inflammation in the neonate.

Most cells in both the innate and adaptive immune system can express IL-10 [43]. IL-10 suppresses inflammation by suppressing various cytokines, including IL-1β and TNFα [44], which is also a mechanism involved in down-regulating hepcidin expression and alleviation of anemia [45]. On the other hand, in sickle cell anemia, iron overload suppresses IL-10, which may contribute to the production of free radicals [46]. However, we observed that WB-iron was negatively correlated with IL-10 levels in all analyses, but not statistically significant, which could be explained by newborns having an impaired IL-10 production compared to adults [47].

### 4.2. Strengths and Limitations

The strengths of the study include a large number of cytokines, adipokines, and other proteins involved in the immune response measured in a large number of newborns (*n* = 398). No previous study has examined the link between early life physiological iron status and the immune system in healthy non-infected neonates. As the study was performed in newborns, extrauterine environmental exposure was unlikely to confound the results, but we cannot rule out any maternal dietary, immunological, or nutritional influences on the newborn. The iron status of the newborn is most likely influenced by the mother’s nutritional iron status, but also maternal and fetal/newborn iron metabolism genetic make-up [48]. Our results could be confounded by other unexamined but associated micro-nutritional factors. Although the newborns were healthy at the time of blood sampling, 50% later developed childhood T1D, which may attenuate the generalizability of the results. Finally, as this is a cross-sectional study, we did not have serial blood tests, and we cannot rule out the risk of reverse causation, for example, that low neonatal IL-6 levels are the cause of higher neonatal WB-Iron content, which could be caused by lower hepcidin levels. 

### 4.3. Future Perspective

During the fetal period and the first years of life, there is rapid development in the immune system. Excessive iron levels have been linked to T1D in both childhood and in adulthood [11,12]; whether these associations are due to early-life adverse immunological changes still remains uncertain. Interestingly, single nucleotide polymorphisms influencing iron status seems to be strongly associated with DNA methylation [49], which may influence immunological pathways [50], and such effects are believed to be long-lasting and not transient. Studies looking at the role of total tissue and circulating iron and immunological health would benefit from a triangulation strategy involving causal epidemiology (Mendelian randomization (MR)), in vitro, and in vivo studies [51]. An MR approach could examine if iron-related single nucleotide polymorphisms [52] were associated with early life immunological alterations, for example, IL-6 and MBL. Further, associated genes could then be knocked out or over-expressed in humanized murine models, and the immunological status could then be examined in infant mice. Different in vitro cell lines could also be exposed to different iron concentrations, for example, IL-6 producing monocytes or hepatocytes, and inflammatory signals, iron chelators, and anti-IL-6-antibodies should also be used in combinations to delineate different effects of iron on IL-6 production.

## 5. Conclusions

In summary, we found that higher neonatal WB-iron content was inversely associated with IL-6 and MBL, which may increase susceptibility to infections. 

## Figures and Tables

**Table 1:** Characteristics of the study sample (including specification of missing values).

Variables	Cases (*n* = 199)	Controls (*n* = 199)
Sex ^1^		
Female, *n*/% of total	103/51.8	95/47.7
Gestational age ^2^,		
Median/interquartile range (IQR), weeks	40.0/1.0	40.0/2.0
Birth weight ^3^,		
Median/IQR, grams	3500/630	3500/744
Maternal age ^4^,		
Median/IQR, years	29.0/6.0	28.0/8.0
Season of blood sampling, *n*/% from total		
Winter Spring Summer Autumn	41/20.6 49/24.6 59/29.6 50/25.1	40/20.1 49/24.6 60/30.2 50/25.1
Period of blood sampling, *n*/% from total		
1991–1993 1994–1998	109/54.8 90/45.2	109/54.8 90/45.2
Human leukocyte antigen (HLA)-risk groups ^5^, *n*/% from total		
High/moderate ^6^ Low/protective ^7^	151/82.5 32/17.5	68/40.0 102/60.0

Missing values: ^1^ 11 controls, ^2^ 1 case and 16 controls, ^3^ 1 case and 17 controls, ^4^ 11 controls, and ^5^ 16 cases and 29 controls. HLA-DQB1 genotype (allele_1/allele_2): ^6^ 03:02/99:99, 03:02/02, 06:04/03:02, 03:01/02, 06:03/03:02, 02/99:99, 06:04/02, 06:04/99:99, 03:01/03:02. 06:04/03:04, and ^7^ 06:02/03:02, 06:02/99:99, 06:02/02, 06:03/99:99, 03:01/99:99, 06:02/03:01, 06:03/03:01, 06:04/03:01, 06:03/02, 03:04/99:99, 03:04/02, 06:02/03:04, 99:99/99:99. NB: 99:99 = remaining alleles.

**Table 2:** Relative change in mean levels of cytokines, adipokines, and other proteins involved in the immune response with 95% confidence bands by 1 unit increase in neonatal whole blood iron content—results from models without correction for multiple testing.

Outcome	Variable	Univariate Model	*p*-value	Multivariate Model	*p*-value
IL-1β	WB-Iron content	1.42 (0.97; 2.07)	0.07	1.37 (0.93; 2.03)	0.11
IL-4	WB-Iron content	1.03 (0.82; 1.30)	0.78	1.05 (0.83; 1.33)	0.69
IL-6	WB-Iron content	**0.62 (0.40; 0.95)**	**0.03**	**0.62 (0.40; 0.95)**	**0.03**
IL-8	WB-Iron content	1.09 (0.89; 1.32)	0.41	1.13 (0.92; 1.39)	0.25
IL-10	WB-Iron content	0.78 (0.43; 1.42)	0.41	0.69 (0.37; 1.27)	0.23
IL-12	WB-Iron content	1.25 (0.90; 1.75)	0.19	1.25 (0.90; 1.75)	0.18
IFNγ	WB-Iron content	1.10 (0.84; 1.45)	0.47	1.09 (0.83; 1.44)	0.53
TNFα	WB-Iron content	0.94 (0.67; 1.32)	0.70	0.99 (0.70; 1.40)	0.95
TGFβ	WB-Iron content	0.92 (0.72; 1.18)	0.52	0.96 (0.76; 1.21)	0.73
Adiponectin	WB-Iron content	1.09 (0.89; 1.34)	0.39	1.12 (0.91; 1.37)	0.28
Leptin	WB-Iron content	0.79 (0.59; 1.05)	0.11	0.85 (0.65; 1.12)	0.26
CRP	WB-Iron content	0.81 (0.56; 1.17)	0.26	0.76 (0.52; 1.12)	0.17
MBL	WB-Iron content	**0.62 (0.41; 0.95)**	**0.03**	**0.63 (0.41; 0.95)**	**0.03**
sTREM-1	WB-Iron content	0.97 (0.69; 1.37)	0.88	0.98 (0.69; 1.39)	0.90

Bold letters indicate significance at a two-sided 5% level. Covariates included in the multivariate models are: neonatal whole blood iron (WB-iron) content, sex, maternal age, and case status (childhood type 1 diabetes (yes/no)). IL, interleukin; IFNγ, interferon gamma; TNFα, tumor necrosis factor alpha; TGFβ, transforming growth factor beta; CRP, C-reactive protein; MBL, mannose-binding lectin; sTREM-1, soluble triggering receptor expressed on myeloid cells-1.

**Table 3:** Relative change in mean levels of cytokines, adipokines, and other proteins involved in the immune response with 95% confidence bands by 1 unit increase in neonatal whole blood iron content—results from models with correction for multiple testing.

Outcome	Variable	Univariate Model	*p*-value	Multivariate Model	*p*-value
IL-1β	WB-Iron content	1.42 (0.81; 2.48)	0.59	1.37 (0.77; 2.44)	0.74
IL-4	WB-Iron content	1.03 (0.74; 1.45)	1.00	1.05 (0.74; 1.49)	1.00
IL-6	WB-Iron content	0.62 (0.33; 1.16)	0.28	0.62 (0.33; 1.16)	0.29
IL-8	WB-Iron content	1.09 (0.81; 1.45)	1.00	1.13 (0.83; 1.53)	0.97
IL-10	WB-Iron content	0.78 (0.32; 1.89)	1.00	0.69 (0.28; 1.70)	0.95
IL-12	WB-Iron content	1.25 (0.77; 2.04)	0.91	1.25 (0.77; 2.04)	0.90
IFNγ	WB-Iron content	1.10 (0.74; 1.65)	1.00	1.09 (0.73; 1.64)	1.00
TNFα	WB-Iron content	0.94 (0.57; 1.55)	1.00	0.99 (0.59; 1.64)	1.00
TGFβ	WB-Iron content	0.92 (0.64; 1.32)	1.00	0.96 (0.68; 1.36)	1.00
Adiponectin	WB-Iron content	1.09 (0.81; 1.48)	1.00	1.12 (0.83; 1.51)	0.98
Leptin	WB-Iron content	0.79 (0.51; 1.21)	0.73	0.85 (0.57; 1.28)	0.97
CRP	WB-Iron content	0.81 (0.47; 1.40)	0.97	0.76 (0.43; 1.35)	0.89
MBL	WB-Iron content	0.62 (0.33; 1.15)	0.28	0.63 (0.34; 1.16)	0.29
sTREM-1	WB-Iron content	0.97 (0.59; 1.61)	1.00	0.98 (0.58; 1.65)	1.00

Covariates included in the multivariate models are: neonatal whole blood iron (WB-iron) content, sex, maternal age, and case status (childhood type 1 diabetes (yes/no)). IL, interleukin; IFNγ, interferon gamma; TNFα, tumor necrosis factor alpha; TGFβ, transforming growth factor beta; CRP, C-reactive protein; MBL, mannose-binding lectin; sTREM-1, soluble triggering receptor expressed on myeloid cells-1.

## Supplementary Materials

The following are available online at https://www.mdpi.com/2072-6643/11/3/543/s1, Figure S1: Associations between log-transformed interleukin 6 and leptin levels and vice versa, Figure S2: Correlogram depicting spearman’s correlation between cytokines, adipokines, and proteins involved in the immune response for the 398 individuals included in this study, Table S1: Absolute levels of cytokines, adipokines, and other proteins involved in the immune response stratified by case status, Table S2: Relative change in mean levels of cytokines, adipokines, and other proteins involved in the immune response with 95% confidence bands by 1 unit increase in neonatal whole blood iron content—results from multivariate models stratified by case status and without correction for multiple testing.
