# Supplementary material for: Association between Neonatal Whole Blood Iron Content and Cytokines, Adipokines, and Other Immune Response Proteins
Source: Nutrients. 2019 Mar 4;11(3):543. doi: 10.3390/nu11030543 (PMC6470999; doi:10.3390/nu11030543)
Supplement: Supplementary file 1 [file nutrients-11-00543-s001.pdf]

Table S1

Absolute levels of cytokines, adipokines and other proteins involved in the immune response stratified by case status

	**Case** (n = 199)	**Control** (n = 199)
IL-1β Median/Q1, Q3, ng/L	175.0/101.5, 252.0	171.0/92.5, 253.5
IL-4 Median/Q1, Q3, ng/L	18.8/12.0, 30.5	17.6/11.9, 31.4
IL-6 Median/Q1, Q3, ng/L	33.0/21.9, 60.7	35.4/20.3, 72.8
IL-8/CXCL8 Median/Q1, Q3, ng/L	188.0/87.5, 296.0	217.0/112.0, 295.0
IL-10 Median/Q1, Q3, ng/L	162.0/82.5, 261.5	172.0/99.5, 252.5
IL-12 Median/Q1, Q3, ng/L	82.3/37.0, 151.7	88.0/47.2, 157.6
IFNγ Median/Q1, Q3, ng/L	41.5/20.3, 69.1	39.9/21.4, 79.4
TNFα Median/Q1, Q3, ng/L	41.3/22.7, 68.8	44.0/26.3, 69.6
TGFβ Median/Q1, Q3, ng/L	929.9/616.5, 1305.7	1018.1/662.3, 1384.1
Leptin Median/Q1, Q3, ng/L	2687.0/1878.0, 3586.0	2932.0/2011.0, 4050.0

Adiponectin<br>Median/Q1, Q3, mg/L	15.7/10.3, 23.9	14.7/10.4, 23.8
CRP<br>Median/Q1, Q3, mg/L	1.1/0.4, 2.2	1.0/0.5, 2.1
MBL<br>Median/Q1, Q3, mg/L	0.8/0.2, 1.3	0.7/0.2, 1.1
sTREM-1<br>Median/Q1, Q3, ng/L	2837.0/1496.6, 5615.7	2791.6/1581.5, 5836.5

*IL, interleukin; IFNγ, interferon gamma; TNFα, tumor necrosis factor alpha; TGFβ, transforming growth factor beta; CRP, c-reactive protein; MBL, mannose-binding lectin; sTREM-1, soluble triggering receptor expressed on myeloid cells-1, CXCL, chemokine (c-x-c motif) ligand; CCL, chemokine (c-c motif) ligand; chemokine IL-8 and CXCL8 are synonyms.

Figure S1

Associations between log-transformed interleukin 6 and leptin levels and vice versa

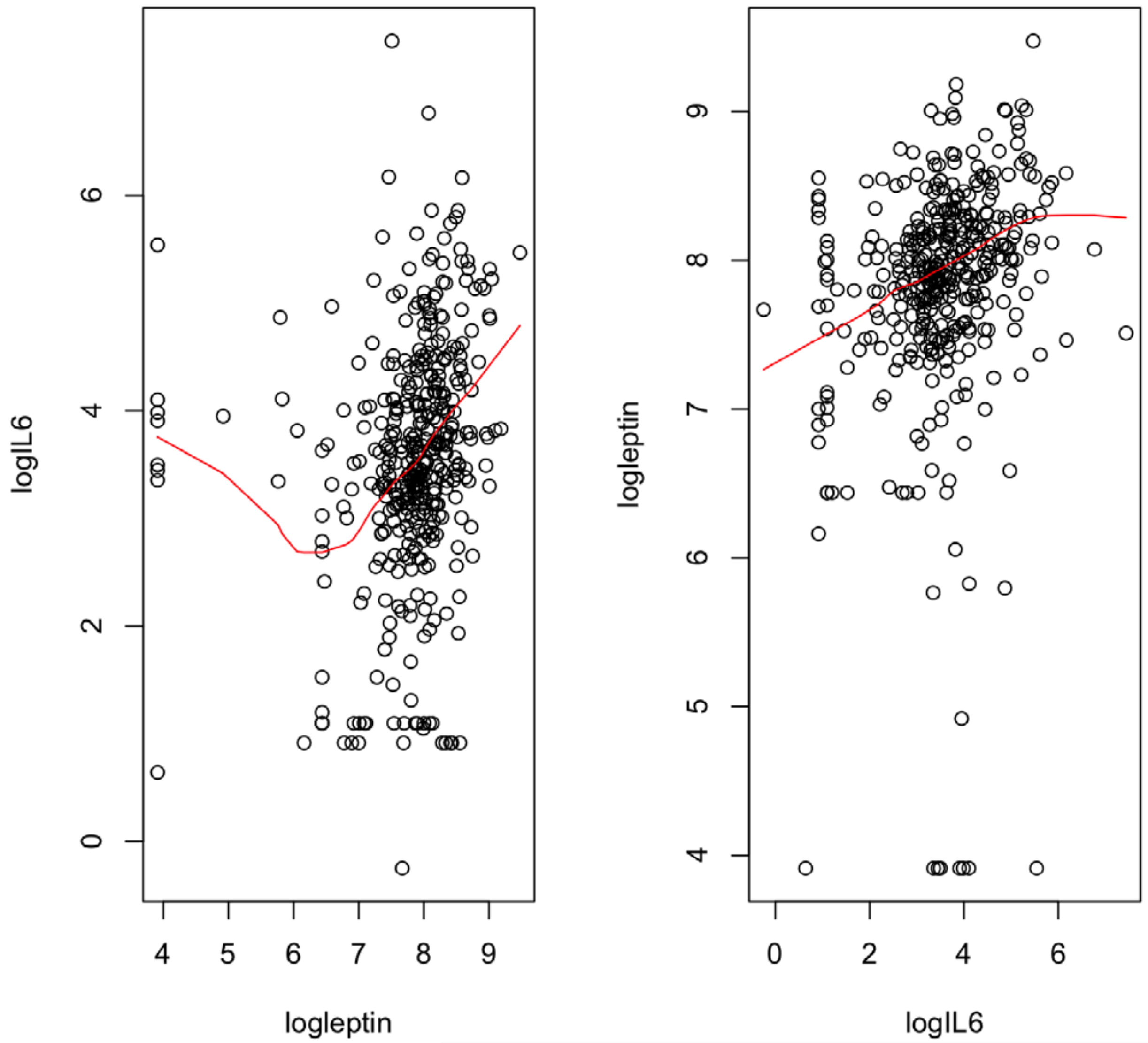

Table S2

Relative change in mean levels of cytokines, adipokines and other proteins involved in the immune response with 95% confidence bands by 1 unit increase in neonatal whole blood iron content—results from multivariate models stratified by case status and without correction for multiple testing.

Outcome	Variable	Cases	P-value	Controls	P-value
IL-1β	WB-Iron content	**2.01 (1.10; 3.67)**	**0.02**	1.14 (0.64; 2.03)	0.67
IL-4	WB-Iron content	1.14 (0.81; 1.61)	0.44	1.02 (0.73; 1.41)	0.92
IL-6	WB-Iron content	0.59 (0.33; 1.07)	0.08	0.67 (0.40; 1.12)	0.13
IL-8	WB-Iron content	1.24 (0.90; 1.72)	0.19	1.10 (0.86; 1.41)	0.45
IL-10	WB-Iron content	0.63 (0.28; 1.48)	0.29	0.75 (0.33; 1.70)	0.49
IL-12	WB-Iron content	1.14 (0.66; 1.99)	0.64	1.38 (0.93; 2.04)	0.11
IFNγ	WB-Iron content	1.02 (0.64; 1.64)	0.93	1.18 (0.86; 1.61)	0.30
TNFα	WB-Iron content	1.05 (0.57; 1.94)	0.87	0.96 (0.64; 1.44)	0.85
TGFβ	WB-Iron content	0.89 (0.60; 1.31)	0.55	0.96 (0.71; 1.31)	0.80
Adiponectin	WB-Iron content	1.10 (0.85; 1.42)	0.47	1.11 (0.82; 1.49)	0.51
Leptin	WB-Iron content	**0.58 (0.36; 0.92)**	**0.02**	1.09 (0.78; 1.51)	0.63
CRP	WB-Iron content	0.71 (0.40; 1.24)	0.23	0.90 (0.56; 1.44)	0.66
MBL	WB-Iron content	0.47 (0.20; 1.11)	0.08	0.76 (0.46; 1.27)	0.30
sTREM-1	WB-Iron content	1.52 (0.85; 2.73)	0.16	0.76 (0.48; 1.20)	0.23

*Bold letters indicate significance at a two-sided 5% level. Covariates included in the multivariate models are: neonatal whole blood iron (WB-iron) content, sex, and maternal age.
IL, interleukin; IFNγ, interferon gamma; TNFα , tumor necrosis factor alpha; TGFβ, transforming growth factor beta; CRP, c-reactive protein; MBL, mannose-binding lectin; sTREM-1, soluble triggering receptor expressed on myeloid cells-1.

Figure S2

Correlogram depicting spearman's correlation between cytokines, adipokines, and proteins involved in the immune response for the 398 individuals included in this study.

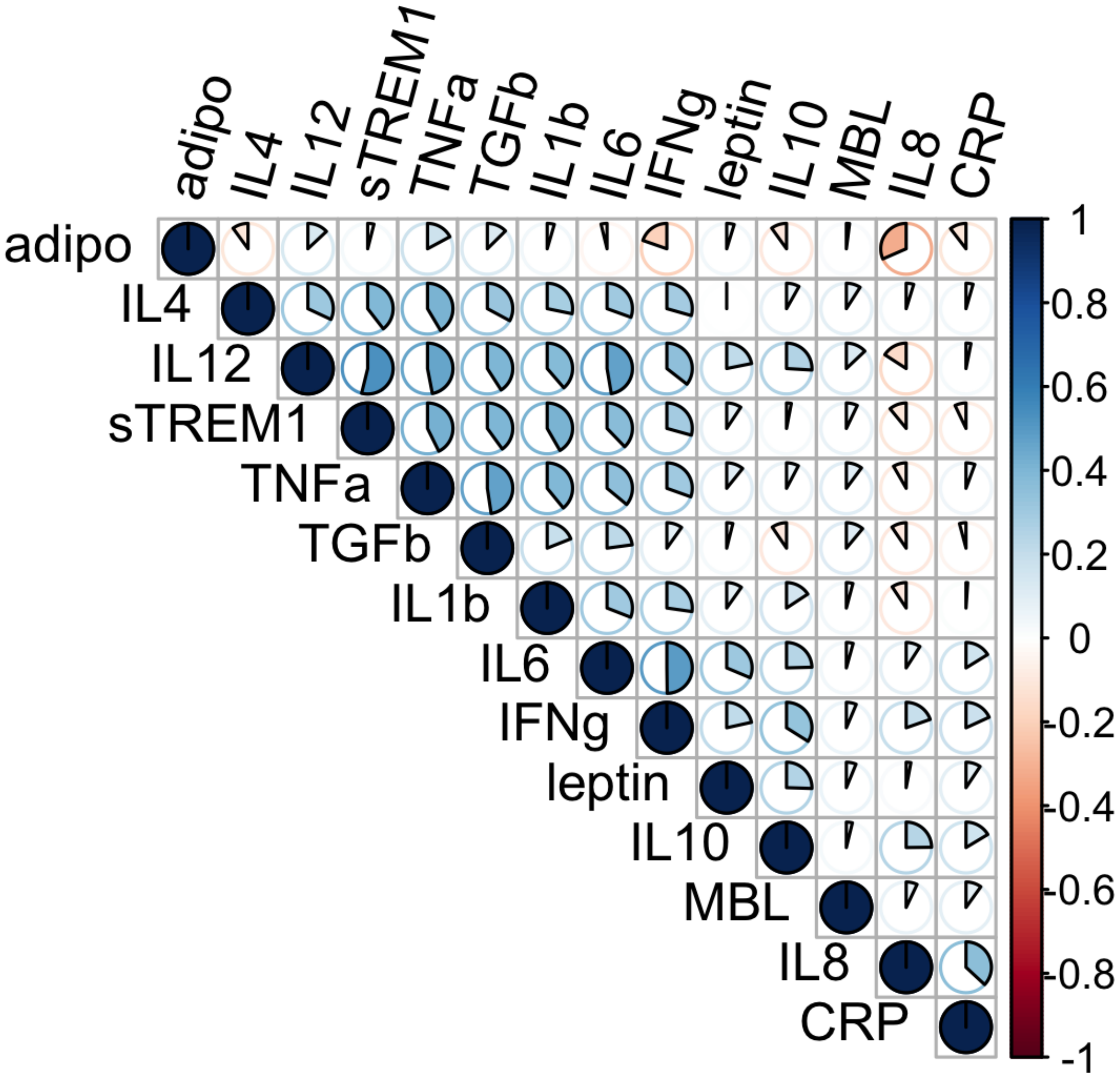

Adipo, adiponectin; IL, interleukin; IL12, IL-12(p70); sTREM1, soluble triggering receptor expressed on myeloid cells-1; TNFa, tumor necrosis factor alpha; TGFb, transforming growth factor beta 1 (active form); IFNg, interferon gamma; MBL, mannose-binding lectin; CRP, c-reactive protein.
